# Adversarial-based latent space alignment network for left atrial appendage segmentation in transesophageal echocardiography images

**DOI:** 10.3389/fcvm.2023.1153053

**Published:** 2023-03-02

**Authors:** Xueli Zhu, Shengmin Zhang, Huaying Hao, Yitian Zhao

**Affiliations:** ^1^Central Laboratory, Department of Ultrasound, Ningbo First Hospital, Ningbo, China; ^2^Cixi Institute of Biomedical Engineering, Ningbo Institute of Materials Technology and Engineering, Chinese Academy of Sciences, Ningbo, China

**Keywords:** left atrial appendage, deep learning, segmentation, transesophageal echocardiography, latent space

## Abstract

Left atrial appendage (LAA) is a leading cause of atrial fibrillation and thrombosis in cardiovascular disease. Clinicians can rely on LAA occlusion (LAAO) to effectively prevent and treat ischaemic strokes attributed to the LAA. The correct selection of the LAAO is one of the most critical stages in the successful surgical process, which relies on the quantification of the anatomical structure of the LAA for successful intervention in LAAO. In this paper, we propose an adversarial-based latent space alignment framework for LAA segmentation in transesophageal echocardiography (TEE) images by introducing prior knowledge from the label. The proposed method consists of an LAA segmentation network, a label reconstruction network, and a latent space alignment loss. To be specific, we first employ ConvNeXt as the backbone of the segmentation and reconstruction network to enhance the feature extraction capability of the encoder. The label reconstruction network then encodes the prior shape features from the LAA labels to the latent space. The latent space alignment loss consists of the adversarial-based alignment and the contrast learning losses. It can motivate the segmentation network to learn the prior shape features of the labels, thus improving the accuracy of LAA edge segmentation. The proposed method was evaluated on a TEE dataset including 1,783 images and the experimental results showed that the proposed method outperformed other state-of-the-art LAA segmentation methods with Dice coefficient, AUC, ACC, G-mean, and Kappa of 0.831, 0.917, 0.989, 0.911, and 0.825, respectively.

## 1. Introduction

Left atrial appendage (LAA) lies anteriorly in the atrioventricular sulcus, which is a finger-like structure extending from the left atrium (LA) with a unique embryonic origin, anatomical structure, and physiological functions (1). With its active contractile and secretory functions, LAA has great significance for relieving the pressure of the left ventricle and ensuring the filling of the left ventricle (2). LAA is the main cause of atrial fibrillation (AF) and thrombosis in cardiovascular disease because of its special anatomical and functional characteristics (3). Thrombus is preferred to form in the LAA and can cause thromboembolic (ischaemic) strokes. In particular, thrombus formed in LAA accounted for 91% of non-valvular AF stroke events and 15–38% of non-AF strokes with cardiomyopathy (4).

Large variability of LAA size and morphology is expected between subjects and previous clinical, and autopsy studies have indicated that LAA size was positively correlated with the risk for stroke and transient ischemic attack (TIA) (5). Moreover, the LAA, with a relatively small orifice, a narrow neck, a multi-lobular structure, and many trabeculations, would further increase the risk for thromboembolic strokes (4). Fortunately, by virtue of catheter radiofrequency ablation surgeries and LAA occlusion (LAAO) can effectively prevent and treat ischemic stroke caused by LAA.

Over the past decade, there has been a dramatical growth in the number of LAAOs (6, 7), and recent studies demonstrated that the correct occluder selection is one of the most critical stages during the successful procedure of LAAO. However, the correct choice of the device for LAAO is a challenging task requiring careful assessment of the highly variable LAA anatomical structure (8, 9): the number, shape, and size of LAA lobes, LAA ostium, determined by the circumflex artery, LAA landing zone (LZ) plane, defined about 10 mm distally from the LAA ostial plane; and (3) LAA depth, measured the distance from the LZ plane to the distal LAA tip. To sum up, the size, shape, and structure of LAA, which are related to AF occurrence and thrombus formation, was proven to be a powerful predictor of ischemic stroke. Meanwhile, the precise grip of LAA morphology for the clinicians is a necessary prerequisite condition for a successful intervention procedure. Therefore, owing to its great clinical significance, it is imperative to accurately identify the morphology of the LAA.

At present, the multi-slice spiral CT (MSCT) and transesophageal echocardiography (TEE) imaging are the most frequently used imaging techniques for LAA (10). MSCTs require contrast injection, have radiation, and can't be used intraoperatively (6). In addition, the LAA is a hollow organ with the feature of changing dimensions due to the increasing LA pressure, and before and during operation procedures the increases in LAA diameters are different among subjects, so preoperative parameter measurements of LAA are not fully representative of intraoperative indicators. TEE is the most commonly recommended imaging modality for procedural guidance and standard device sizing during the LAAO operation by the device manufacturers (10, 11). However, there are still some inherent disadvantages of TEE imaging, namely: the inter- and intra-observer variability resulting from the manual estimation of ultrasonic images; the correct identification of the LAA shape needs a certain amount of experience, especially when the images are interfered with by artifacts. Thereby, it is imperative to enhance the intelligence and automation of the TEE image identification and improve the repeatability and short learning curve for a starting operator.

With the recent advances in artificial intelligence, it has become possible to automatically identify the size, shape, and structure of LAA (6). Previous research developed image-processing techniques to segment the target anatomy for echo datasets, using simple image-based techniques, deformable models, or machine-learning strategies (12). Simard et al. (7) provided an image-based technique applied in TEE images, extracting realistic LAA shapes but being extremely time-demanding due to the high number of manual corrections required. Pedro Morais et al. presented a semi-automatic solution to complete the image segmentation and acquire relevant clinical measurements (9). However, previous manual or semi-automatic segmentation of the LAA based on ultrasound data is slow and time-consuming, and interpretation greatly varies among expert users. Thus, an approach for accurate automated segmentation of the LAA should be proposed. Indeed, taking into consideration the state-of-the-art, efficient and automated strategies to segment the LAA in TEE images were, to our best knowledge, not described, particularly due to (1) the complex curvilinear and tubular anatomical shape of the LAA; (2) the high anatomical variability of this structure; (3) the low image quality. Therefore, a precise, more effective, and fully automated LAA segmentation system in TEE images with less impact from human error is needed.

In this paper, we propose a deep learning-based LAA segmentation network for TEE images that enhances segmentation performance by introducing prior knowledge of the label. The proposed method consists of three parts: an LAA segmentation network, a label reconstruction network, and a joint latent space alignment loss. Firstly, we utilize the ConvNeXt as the backbone of the segmentation and reconstruction network to enhance the feature extraction ability of the network. Furthermore, the label reconstruction network encodes the shape prior features in the LAA labels in the latent space vector. A latent space alignment loss combining adversarial-based loss and contrast learning loss aligns the latent space of the reconstructed network with that of the segmentation network. It aims to improve LAA segmentation accuracy by introducing the shape prior to the segmentation network. The experimental results show that our method achieves state-of-the-art performance in LAA segmentation, can effectively extract accurate LAA structures, and can assist in improving the accuracy of thrombosis diagnosis and the successful performance of LAAO.

## 2. Related work

### 2.1. Left atrial appendage segmentation

In the last decade, many LAA segmentation and detection methods have been proposed to assist in LAA occlusion. Wang et al. (13) proposed a non-model semi-automatic method for LAA segmentation based on Computed Tomography Angiography (CTA) images. The method relied on the manual selection of four datum points to obtain the LAA bounding box and used parametric max-flow generation and random forest to segment 2-D LAA slices merged into the 3D model. Zheng et al. (14) proposed a fully automated system for segmentation of the left atrium based on computed tomography (CT) images, including the ventricle, LAA, and pulmonary veins. A multi-local shape prior model was introduced to model the left atrium, and the experiments on 687 CT images demonstrate the robustness and advancement of the method. Qiao et al. (15) proposed a joint atlas-optimized segmentation method to segment the left atrium, pulmonary veins, and LAA from magnetic resonance angiography (MRA) images. The method formulated the segmentation as a single registration problem between a given image and all atlas images and used level sets to refine the atlas-based segmentation. With the widespread application of deep learning techniques in medical image segmentation tasks, the LAA segmentation methods based on deep learning have been further developed. For example, Jin et al. (1) proposed an LAA segmentation method based on a fully convolutional neural network and conditional random fields in CTA images. The network segmented the LAA in each 2D slice of a manually provided bounding box and then used a 3D conditional random field to merge the segmented 2D probability maps into the final 3D volume. For TEE imaging, Ghayoumi Zadeh et al. (16) used the You Only Look Once (YOLO) algorithm to detect the LAA region. However, the algorithm can only detect rectangular areas of LAA and fails to obtain pixel-level structure information. In this paper, we present an effective, deep learning-based pixel-level LAA segmentation framework for TEE images. Considering the complex shape knowledge of the LAA, the proposed method utilizes the prior knowledge stored in the latent space of the mask to guide the segmentation network to output accurate LAA edges.

### 2.2. GAN-based medical image segmentation

With the first application of Generative Adversarial Networks (GANs) in image segmentation (17), GANs have been widely used in medical image segmentation tasks, effectively improving the accuracy of medical image segmentation. The current performance of GANs to assist in segmentation tasks is 2-fold: (1) data augmentation to improve the generalization of segmentation networks; (2) adversarial loss to optimize the distance between predicted results and labels. Data augmentation-based segmentation methods mainly employ GANs to synthesize target images for assisting fully/weakly supervised segmentation networks. For example, Conte et al. (18) utilized GAN to synthesize missing MRI sequences and demonstrated that the images generated by GAN can effectively improve the precision of the segmentation network. Chen et al. (19) leveraged a generation network of unpaired CT-MRI data to assist MRI images for the craniomaxillofacial segmentation framework. The network involved a cross-modality image synthesis model in learning the mapping between CT and MRI and an MRI segmentation model. Iqbal and Ali (20) proposed a new medical imaging generative adversarial network (MI-GAN). MI-GAN generated synthetic retinal images and the corresponding segmentation masks to assist in the retinal image segmentation task. The adversarial loss-based segmentation method uses generative adversarial networks to generate segmentation results that are indistinguishable from manual segmentation. Moeskops et al. (21) introduced an additional adversarial loss function to improve the CNN-based MRI image segmentation network, which can motivate the network to generate high-quality segmentation results. Yang et al. (22) proposed an automatic GAN-based segmentation algorithm for liver extraction from 3D CT volumes. The network used an encoder-decoder structure integrated with multi-level feature concatenation and deep super-vision for liver segmentation. Dong et al. (23) proposed a segmentation network based on conditional generative adversarial networks for left ventricle segmentation on 3D echocardiography. The network facilitated the fusion of large 3D spatial contextual information from 3D echocardiography by self-learning structured loss. Wang et al. (24) proposed a patch-based unsupervised domain-adaptive optic disc and optic cup segmentation framework, which addresses the domain transfer challenge by aligning the target domain's segmentation results with the source domain's segmentation results. The network presented a new morphology-aware segmentation loss to guide the network in generating accurate and smooth segmentations. Unlike the above methods that introduce adversarial loss at the network's output, the proposed method introduces adversarial loss into the latent space layer of the network for constraint. It can facilitate the segmentation network to learn the contextual information of the labels to improve the accuracy of the overall segmentation results and the model's generalization ability.

## 3. Method

**Figure 1:**
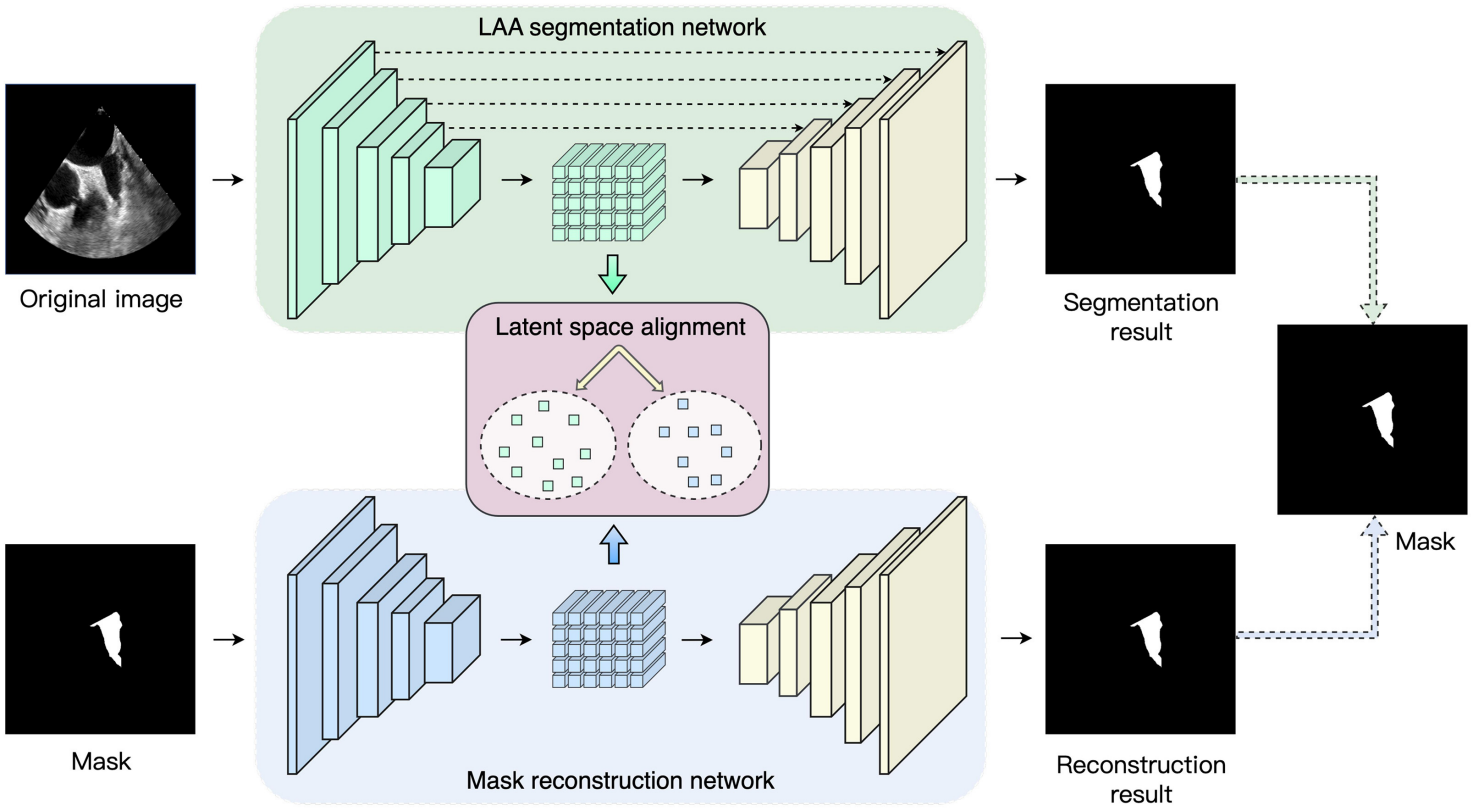
Overview of the proposed network. The framework consists of three parts: LAA segmentation network, maks reconstruction network and latent space alignment.

The overall framework proposed in the paper is shown in Figure 1. In the training process, the proposed method consists of three parts: LAA segmentation network, mask reconstruction network, and latent space alignment loss. First, we train a mask auto-encoder reconstruction network based on the ConvNeXt model to obtain the latent space feature of the mask. We then construct an LAA segmentation network and adopt generative adversarial learning to align the segmentation network's latent space with the mask reconstruction's latent space vector, thus introducing prior knowledge of the LAA mask. In addition, we introduce contrast learning loss to enhance the association between the prior latent features and the latent space features of the original image to increase the LAA segmentation accuracy.

### 3.1. LAA segmentation network

A U-shape structure is employed to segment the LAA and consists of an encoder, a decoder, and skip connection layers. The encoder enables the extraction of shallow and deep features of the fused image to obtain the latent space, after which the decoder is utilized to recover the mask prediction results from the latent space. The skip connection layers fuse the feature map at each stage of the encoder with the feature map obtained by upsampling the decoder layers. This allows the decoder to access high-level features learned by the encoder and helps the decoder to accurately preserve the details of the input image.

**Figure 2:**
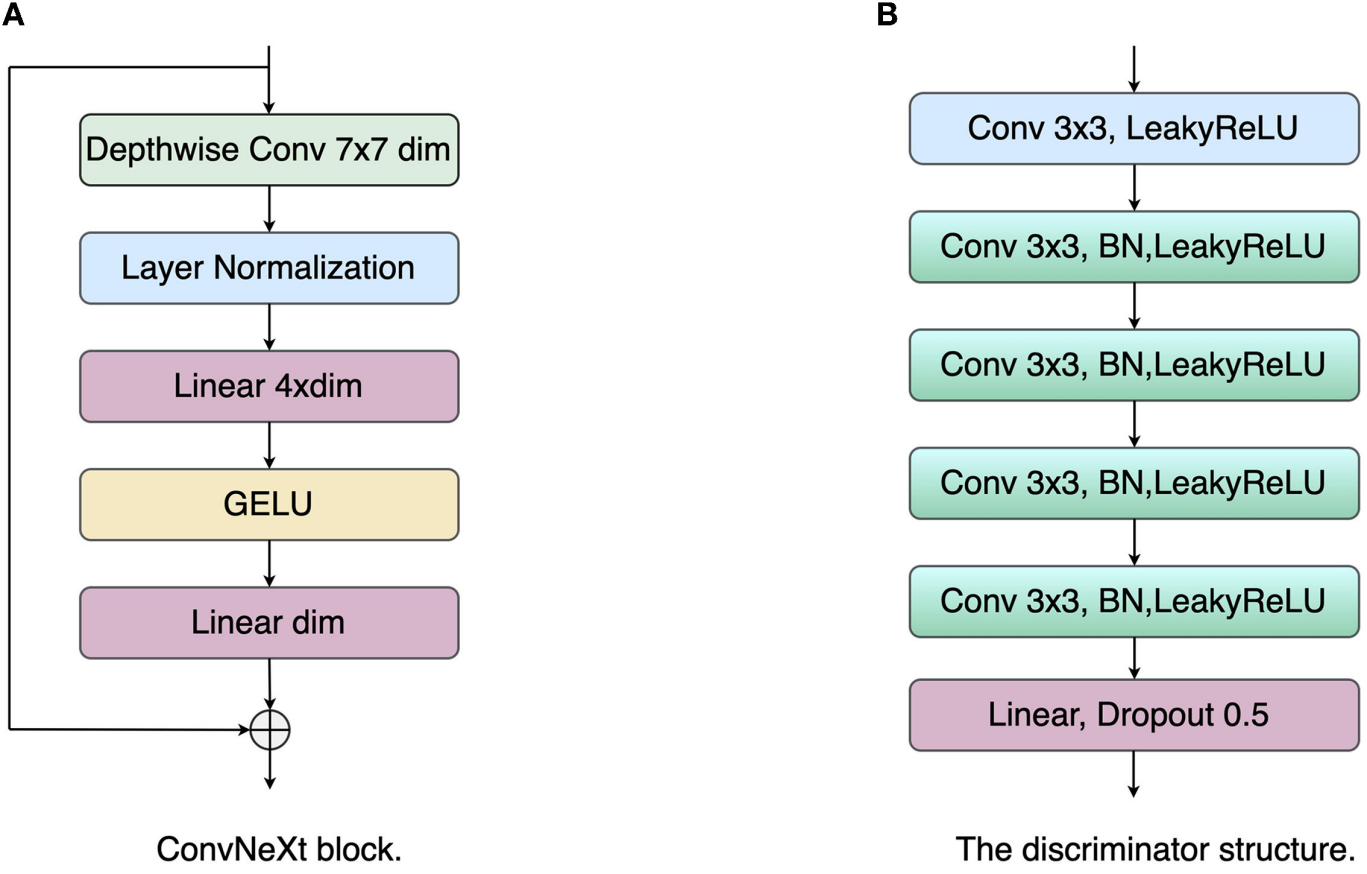
The structure illustrations of the ConvNeXt block and discriminator. **(A)** ConvNeXt block; **(B)** Discriminator network.

In general, the encoder structure in the U-Net model is a VGG network (25), i.e., each layer consists of two 3 × 3 convolutional kernels, a linear rectification function (ReLU), and a max-pooling layer with a step size of 2. In recent years, with the successive introduction of ResNet (26), DenseNet (27), and Transformer (26), the feature extraction capability of neural networks for input images has been significantly improved. In order to extract richer feature information, the ConvNeXt (28) network is used as the base network of the encoder. ConvNeXt uses ResNet50 as the base network and carries out macroscopic design and improvement of the network to enhance the feature extraction capability. First, ConvNeXt adjusts the number of blocks in each stage of ResNet-50 to (3, 3, 9, 3). In the first layer of the network, the Stem convolution layer in the Transformer with a step size of 4 and a kernel size of 4 is employed to downsample the input image. In each convnext block, the network introduces a deeply separable convolution module, which operates the 3 × 3 convolution in channels and then performs channel fusion by 1 × 1 convolution. Subsequently, an inverse bottleneck layer is adopted to avoid information loss. As shown in Figure 2, the convolution channel numbers are large in the middle and small in the ends: *dim*, *dim*×4, *dim*, with large convolution kernels 7 × 7 introduced into the block to enhance the spatial feature extraction ability, where *dim* represents the dimension of the input feature. In addition, the network is optimized with some network details, including normalization layers, activation functions, and downsampling. Specifically, the network chooses to replace RELU with GELU as the activation function. It draws on the Transformer concept and uses a less activation function strategy: the GELU activation function is added between the two 1 × 1 convolution layers, as shown in Figure 2. For the normalization layer, the network replaces the Batch Normalization (BN) layer with the Layer Normalization (LN) layer, using a less normalization layer strategy: an LN layer is added between the two 1 × 1 convolutions. For the downsampling operation, ConvNeXt adopts a 2 × 2 convolution with a step size of 2 inserted between the different stages. Finally, an LN layer is added before and after the downsampling layer and after the Global Average Pooling (GAP) layer to maintain the model stability. For each decoder layer, we use a residual module and an upsampling module to recover the input mask information. During this training process, cross-entropy loss and dice coefficient loss $L_{Dice}$ are used to optimize the LAA segmentation network:


(1) $$\begin{array}{l} L_{CE}=-\overset{C}{\underset{i=1}{\sum }}g_{i}\log p_{i} \end{array}$$

(2) $${ℒ}_{Dice}=1-\frac{2\sum_{i=1}^{N}p_{i}g_{i}+\epsilon }{\sum_{i=1}^{N}p_{i}^{2}\sum_{i=1}^{N}g_{i}^{2}+\epsilon }$$

Where N is the number of all pixels, *p*_*i*_ and *g*_*i*_ represent the i-th pixel of the prediction map and the ground truth, respectively. C = 2 indicates the number of classes, and ϵ is a small positive constant used to avoid numerical problems and accelerate the convergence of the training process.


(3) $$\begin{array}{l} L_{seg}=\lambda_{CE}\cdot L_{CE}+\lambda_{Dice}\cdot L_{Dice} \end{array}$$

Where λ_*CE*_ and λ_*Dice*_ are set to 1 and 1 empirically.

### 3.2. Mask reconstruction network

We adopt auto-encoder structure as mask reconstruction network, including an encoder and a decoder. In the mask reconstruction network, we also employ ConvNeXt as the encoder with the residual module and the upsampling layer forming the decoder layer. During training, we use L1 loss to optimize the reconstruction results, which can be represented as:


(4) $$\begin{array}{l} L1=\overset{N}{\underset{i=1}{\sum }}|g_{i}-r_{i}| \end{array}$$

Where *r*_*i*_ and *g*_*i*_ represents the i-th pixel of the reconstruction map and the LAA mask, respectively.

### 3.3. Latent space alignment loss

The latent space alignment loss is the main contribution of the paper. Unlike the classical pixel-level loss, the latent space alignment loss allows optimization of the segmentation results in terms of high-dimensional feature alignment: the extraction of the shape prior knowledge in the encoder is enhanced by constraining the consistency of the label with the image in the latent space. Most works optimize the network for pixel-level losses, ignoring the contribution of intermediate-layer features to the network. In addition, the skip connection layer of the U-shape network tends to pass multi-scale information from the encoder to the decoder. This can lead to the network tending to use low-level encoder layer features while ignoring high-level encoder features. However, these low-level encoder features have insufficient contextual information. The network needs to force the encoder layer to output high-level encoder features to provide global information to the decoder. Therefore, we propose a latent space alignment loss, including an adversarial loss and a contrast learning loss. First, for the adversarial loss, we used the least squares generative adversarial network (LSGAN) (29) to perform feature alignment between the latent space vector of the original image and that of the segmentation result. As shown in Figure 2, the discriminator network consists of five convolutional layers with a step size of 1 and a kernel size of 3, and a fully connected (FC) layer. Each convolutional layer is followed by a LeakyReLU layer with a slope of 0.2 and a batch normalization layer, and the network outputs the final result through the FC layer. The objective function of LSGAN is as follows:


(5) $$\begin{array}{l} \min_{D}{ℒ}_{\text{GAN}}(D)=\frac{1}{2}E_{x\sim p_{\text{data}}(k)}\left[{\left(D(k)-1\right)}^{2}\right]\\ \;+\frac{1}{2}E_{x\sim p_{x}(x)}\left[{\left(D(G(x))\right)}^{2}\right], \end{array}$$

(6) $$\min_{G}{ℒ}_{\text{GAN}}(G)=\frac{1}{2}E_{x\sim p_{x}(x)}\left[{\left(D(G(x))-1\right)}^{2}\right]$$

Where *D* and *G* represent the discriminators and encoders of the segmentation network, *k* represents the latent space features of the mask, and *x* represents the original TEE image.

Nevertheless, the GAN-based alignment loss tends to align the overall marginal distribution from the two groups of features and may ignore the differences in the latent space features of different input images within a batch. Therefore, based on the adversarial loss, we introduce a contrast learning loss to enhance the similarity of the pairwise latent space features. The contrast learning loss can be defined as:


(7) $${ℒ}_{CL}=-\log\frac{\exp\left(q\cdot k_{+}/\tau \right)}{\sum_{i=0}^{B}\exp\left(q\cdot k_{i}/\tau \right))}$$

Where *q* represents the latent space feature from the original image, and *k*_+_ is the corresponding latent space features from the mask for *q*, denoting positive samples. *B* represents the number of samples in a batch. τ represents the temperature coefficient to adjust the level of attention to difficult samples, and we set τ to 0.1. Thus, the joint latent space alignment loss $L_{LA}$ is defined as:


(8) $$\begin{array}{l} L_{LA}=\lambda_{GAN}\cdot L_{GAN}+\lambda_{CL}\cdot L_{CL} \end{array}$$

Where λ_*GAN*_ and λ_*CL*_ are set to 1 and 1 empirically.

## 4. Experiment

### 4.1. Dataset

In this paper, we constructed an echocardiography left atrial appendage segmentation dataset. The local dataset was acquired by Philips Epiq 7c (Philips Ultrasound, Bothell, USA) or a Philips IE33 scanner from the ultrasound department of Ningbo First Hospital. It includes 1,783 images from 41 patients (containing 18 non-thrombotic and 23 thrombotic LAA patients). All the images have the same image resolution of 800 × 600. We randomly assigned 1,783 images to the training, testing and validation sets in a ratio of 4:1:1. We ensure that TEE images from one patient fall into the same training, validation, or testing set. Two experts were invited to label the boundaries of LAA regions manually for all 1,783 images, and their consensus was finally used as ground truth.

### 4.2. Implementation details

The proposed method was implemented by the publicly available Pytorch Library in the NVIDIA GPU (GeForce RTX 3090 with 24 GB). In the training phase, we employed an Adam optimizer (30) to optimize the deep model. We used a gradually decreasing learning rate, starting from 0.0001, and a momentum of 0.9. In each iteration. We resized the image to 448 × 448 for training, and the batch size was set to 8 during the training. In addition, online data enhancement with a random rotation from −10° to 10° was employed to enlarge the training set.

### 4.3. Evaluation metrics

To achieve comprehensive and objective assessment of the segmentation performance of the proposed method, the following metrics are calculated and compared: the following metrics are calculated and compared:

- Area Under the ROC Curve (AUC);
- Accuracy (ACC) = (TP + TN) / (TP + TN + FP + FN);
- *G*−*mean* score = $\sqrt{Sensitivity\times Specificity}$;
- Dice coefficient (Dice) = 2 × TP / (FP + FN + 2 × TP);
- Intersection over union (IOU) = TP / (FP + FN + TP);
- *Kappa* score = (*Accuracy*−*p*_*e*_)/(1−*p*_*e*_).

Where TP is true positive, FP is false positive, TN is true negative, and FN is false negative. *p*_*e*_ in *Kappa* score represents opportunity consistency between the ground truth and prediction, and it is denoted as:


(9) $$\begin{array}{l} p_{e}=((TP+FN)(TP+FP)+(TN+FP)(TN+FN))\\ \;/(TP+TN+FP+FN{)}^{2} \end{array}$$

### 4.4. Performance comparison and analysis

To demonstrate the segmentation performance of the proposed method, several state-of-the-art segmentation methods are introduced for comparison: U-Net (31), ResU-Net (32), CE-Net (33), SwinU-Net (34), and TransU-Net (35). U-Net and ResU-Net are benchmark models for medical image segmentation, and CE-Net, SwinU-Net, and TransU-Net are the advanced medical image segmentation methods. Therefore, comparing these classical methods, the segmentation performance of the proposed method can be effectively demonstrated. Table 1 shows the comparative results of different segmentation methods for LAA segmentation. Firstly, the proposed method achieves state-of-the-art performance in all metrics. In particular, the proposed method achieved LAA segmentation with Dice, IOU, AUC, ACC, G-mean, and Kappa of 0.831, 0.724, 0.917, 0.989, 0.911, and 0.825, respectively, which indicates that the segmentation results of the proposed method are in good agreement with the expert annotations. Table 2 shows the LAA segmentation results in the thrombus and non-thrombus groups using different segmentation methods. For the non-thrombotic group, all comparative segmentation methods achieve advanced performance. The proposed method slightly underperformed CE-Net and TransU-Net in Dice and Kappa metrics, and achieved the best performance in all other metrics. The segmentation performance of thrombus group performed poorly compared to the segmentation results of the non-thrombotic group. Among them, the Dice for all comparison methods failed to exceed 0.8, which indicates that thrombus segmentation is very challenging. The segmentation performance of our method in the thrombus group far exceeds that of the other compared methods. Specifically, our method improved each metric compared to CE-Net and TransU-Net: Dice improved by 2.0% and 2.3%, AUC improved by 2.9% and 2.1%, G-mean improved by 3.5% and 2.5%, and Kappa improved by 3.1% and 3.4%, respectively. This suggests that the proposed method not only achieves comparatively advanced segmentation performance in the non-thrombus group, but also enables better segmentation of thrombus in TEE images. This also demonstrates the proposed approach has great advantages in LAA segmentation and can alleviate the problem of hard-to-segment thrombus samples.

**Table 1:** LAA segmentation performance of comparison methods on TEE images.

**Method**	**Dice**	**AUC**	**ACC**	**G-mean**	**IOU**	**Kappa**
U-Net	0.778 ± 0.135	0.901 ± 0.071	0.986 ± 0.009	0.893 ± 0.082	0.654 ± 0.161	0.770 ± 0.138
ResU-Net	0.805 ± 0.134	0.914 ± 0.070	0.987 ± 0.010	0.907 ± 0.079	0.691 ± 0.156	0.799 ± 0.137
CE-Net	0.819 ± 0.112	0.907 ± 0.071	0.988 ± 0.008	0.898 ± 0.082	0.706 ± 0.142	0.813 ± 0.115
SwinU-Net	0.807 ± 0.130	0.893 ± 0.077	0.988 ± 0.008	0.881 ± 0.102	0.693 ± 0.156	0.801 ± 0.132
TransU-Net	0.809 ± 0.129	0.897 ± 0.070	0.988 ± 0.010	0.887 ± 0.081	0.696 ± 0.152	0.803 ± 0.131
Our method	**0.831** **±** **0.113**	**0.917** **±** **0.066**	**0.989** **±** **0.007**	**0.911** **±** **0.075**	**0.724** **±** **0.140**	**0.825** **±** **0.116**

The values in bold represent the best of all the comparative experimental results.

**Table 2:** LAA segmentation performance of the comparative methods for the non-thrombus and thrombus groups.

	**Non-thrombus**				**Thrombus**			
**Method**	**Dice**	**AUC**	**G-mean**	**Kappa**	**Dice**	**AUC**	**G-mean**	**Kappa**
U-Net	0.860 ± 0.079	0.950 ± 0.037	0.948 ± 0.040	0.855 ± 0.081	0.741 ± 0.139	0.879 ± 0.072	0.868 ± 0.085	0.733 ± 0.142
ResU-Net	0.863 ± 0.075	0.957 ± 0.045	0.859 ± 0.076	0.859 ± 0.076	0.778 ± 0.146	0.895 ± 0.070	0.886 ± 0.081	0.772 ± 0.149
CE-Net	0.881 ± 0.066	0.961 ± 0.028	0.960 ± 0.029	0.877 ± 0.068	0.778 ± 0.135	0.867 ± 0.077	0.852 ± 0.099	0.771 ± 0.137
SwinU-Net	0.869 ± 0.089	0.949 ± 0.037	0.947 ± 0.041	0.865 ± 0.091	0.784 ± 0.122	0.872 ± 0.074	0.858 ± 0.090	0.777 ± 0.124
TransU-Net	**0.884** **±** **0.060**	0.945 ± 0.041	0.942 ± 0.045	**0.881** **±** **0.062**	0.775 ± 0.137	0.875 ± 0.070	0.862 ± 0.082	0.768 ± 0.140
Our method	0.880 ± 0.080	**0.966** **±** **0.024**	**0.965** **±** **0.025**	0.877 ± 0.081	**0.808** **±** **0.120**	**0.896** **±** **0.067**	**0.887** **±** **0.078**	**0.802** **±** **0.122**

The values in bold represent the best of all the comparative experimental results.

**Figure 3:**
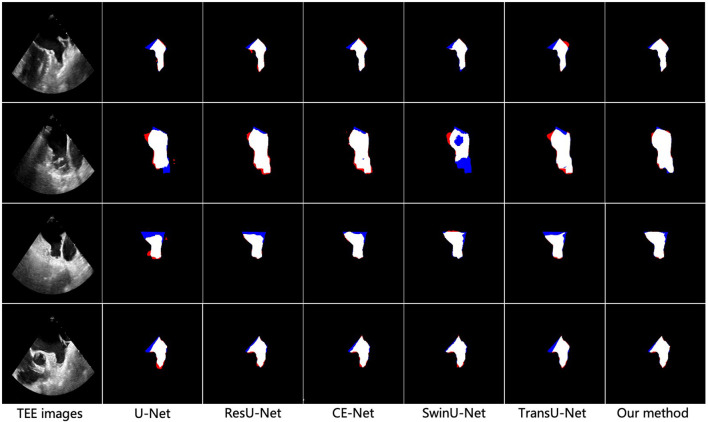
Visualization results of different methods for vessel segmentation on TEE images. From left to right: the original image, the vessel segmentation results obtained by U-Net, ResU-Net, CS-Net, SwinU-Net, TransU-Net, and the proposed method, respectively. Blue represents under-segmentation and red indicates over-segmentation.

To better demonstrate the superior performance of the proposed method on LAA segmentation, we visualize the segmentation results of all compared methods. Figure 3 illustrates the segmentation results for the four test samples, where the first and second rows show the TEE images with non-thrombotic and the corresponding LAA segmentation results, and the third and fourth rows show the TEE images with thrombotic and the corresponding LAA segmentation results. From observing the segmentation results in Figure 3, it can be seen that the comparative methods have more over- and under-segmentation in their segmentation maps, while the proposed method produces smoother and more accurate LAA regions. Furthermore, the segmentation visualization results of CE-Net and TransU-Net are second only to the proposed methods, in line with the performance of the metrics in the Table 1. Specifically, all comparison methods appear severely under-segmented (blue), which is highly detrimental to measuring LAA shape. Under-segmentation and over-segmentation can damage the LAA morphological structure, which is detrimental to the successful progress of the blocking procedure. In addition, it can be observed from Figure 3 that most of the segmentation results show poor edge detail at the LAA opening and the comparison methods cannot correctly identify the location of key points at the LAA opening. It indicates that these segmentation methods have difficulty capturing the contextual information of TEE images and the lack of prior clinical leads to incorrect segmentation of critical edges. The proposed method can accurately capture prior clinical knowledge and effectively improve the segmentation performance of edges.

### 4.5. Ablation studies

In this paper, our proposed method employs three modules to build the LAA segmentation framework, i.e., ConvNeXt block, GAN loss, and contrast loss. To evaluate the effectiveness of each module, we validate the segmentation performance on the local TEE dataset using different combinations of these modules.

#### 4.5.1. Ablation for ConvNeXt backbone

To discuss the performance of the ConvNeXt backbone, we compared the original U-Net with our proposed encoder-decoder architecture (with ConvNeXt as the backbone), as shown in Table 3. Compared to the original U-Net, our encoder-decoder architecture with the ConvNeXt backbone achieves better performance on AUC, ACC, Kappa, Dice, and FDR. This demonstrates the advantage of the ConvNeXt backbone in feature extraction.

**Table 3:** Ablation studies of our segmentation method in TEE images.

**ConvNeXt block**	**GAN loss**	**Contrast loss**	**Dice**	**AUC**	**ACC**	**Gmean**	**IOU**	**Kappa**
			0.778 ± 0.135	0.901 ± 0.071	0.986 ± 0.009	0.893 ± 0.082	0.654 ± 0.161	0.770 ± 0.138
✓			0.815 ± 0.112	0.899 ± 0.067	0.988 ± 0.008	0.890 ± 0.078	0.701 ± 0.141	0.809 ± 0.115
✓	✓		0.827 ± 0.127	0.903 ± 0.070	**0.989** **±** **0.007**	0.894 ± 0.084	0.721 ± 0.153	0.822 ± 0.129
✓	✓	✓	**0.831** **±** **0.113**	**0.917** **±** **0.066**	**0.989** **±** **0.007**	**0.911** **±** **0.075**	**0.724** **±** **0.140**	**0.825** **±** **0.116**

The values in bold represent the best of all the comparative experimental results.

#### 4.5.2. Ablation for GAN loss

We performed GAN loss-based learning of latent space alignment using a ConvNeXt backbone-based segmentation network and a label reconstruction network to demonstrate the effect of GAN loss in the LAA segmentation network. Table 3 shows the comparative performance between the network based on the ConvNeXt backbone and the network incorporating the ConvNeXt backbone and GAN loss. We can observe that GAN loss-based latent space alignment learning achieved higher scores in Dice, AUC, ACC, G-mean, IOU, and Kappa than segmentation network using only the ConvNeXt backbone. This suggests that GAN loss-based latent space alignment learning can improve the segmentation performance of LAA edge details by introducing the prior shape knowledge.

#### 4.5.3. Ablation for contrast loss

Furthermore, we analyze the effect of contrast loss in latent space alignment learning on LAA segmentation performance. In latent space alignment learning, the segmentation results based on GAN loss learning are regarded as the initial segmentation results; the final LAA segmentation results are derived from the joint GAN loss and contrast loss. Therefore, we compared the preliminary segmentation results with the final LAA segmentation results of the joint loss. As shown in Table 3, the final LAA segmentation performance improved in most metrics compared to the results of the coarse stage. Compared to using only GAN loss, the segmentation performance of joint loss increased in most metrics: Dice improved by 0.4%, AUC by 1.4%, G-mean by 1.7%, IOU by 0.3%, and Kappa by 0.3%. It demonstrates that the joint loss can close the label's latent space to the corresponding image's latent space, which yields more accurate prior knowledge and thus improves the LAA segmentation performance.

## 5. Conclusion

The LAA size, shape, and structure are associated with the development of atrial fibrillation and thrombus formation and have been shown to be powerful predictors of ischaemic stroke. The LAA occlusion (LAAO) can effectively prevent and treat ischaemic strokes caused by the LAA. The size and shape of the LAA vary considerably between subjects, which can present challenges for correctly selecting the occluder. Therefore, the clinician's precise knowledge of the LAA morphology is a prerequisite for successful interventional surgery. In order to better characterize the morphological structure of the LAA, we require a more accurate technique to segment the LAA region. In this paper, we propose a deep learning-based LAA segmentation network in TEE images, including three components: LAA segmentation network, label reconstruction network, and latent space alignment loss. Firstly, we use the ConvNeXt module as the backbone of the segmentation and reconstruction network to enhance the feature extraction capability of the network. The label reconstruction network can encode the shape prior to the LAA mask in the latent space. The latent space alignment loss is introduced prior to the LAA segmentation network to improve the accuracy of LAA edge segmentation. The experimental results show that our method surpasses other advanced comparison methods in LAA segmentation and can effectively extract accurate LAA structures to improve thrombus diagnosis accuracy and successful LAAO.

Although the proposed method has stressed its potential for LAA segmentation of TEE images, several aspects still need to be improved. On the one hand, the performance of the proposed method for LAA segmentation in non-thrombotic patients is significantly better than that of LAA segmentation in thrombotic patients. This implies that the morphological structure of the LAA in TEE images with thrombus is relatively complex, and the existing segmentation method is not yet able to meet the clinical requirements and needs further improvement. On the other hand, our method only performs LAA segmentation on 2D TEE images and cannot capture 3D spatial features. It is not conducive to the next step of LAA reconstruction and the measurement of morphological structures. In the future, we will extend the proposed method to LAA segmentation of 3D TEE images, allowing for LAA reconstruction and morphological classification.

## Data availability statement

The original contributions presented in the study are included in the article/supplementary material, further inquiries can be directed to the corresponding author.

## Ethics statement

The studies involving human participants were reviewed and approved by the Ethics Committees of Ningbo Institute of Industrial Technology, Chinese Academy of Sciences. The patients/participants provided their written informed consent to participate in this study.

## Author contributions

XZ and SZ carried out this study, performed the research, and wrote the manuscript. HH designed this study, analyzed the data, and revised the manuscript. YZ developed the computational pipeline and revised the manuscript. All authors read, edited, and approved the final manuscript.
